# Instruments to measure patient experience of healthcare quality in hospitals: a systematic review

**DOI:** 10.1186/s13643-015-0089-0

**Published:** 2015-07-23

**Authors:** Michelle Beattie, Douglas J. Murphy, Iain Atherton, William Lauder

**Affiliations:** School of Health Sciences, Centre for Health Science, University of Stirling, Highland Campus, Old Perth Rd, Inverness, IV2 3JH UK; Quality, Safety and Informatics Research Group, University of Dundee, Dundee, UK; School of Nursing, Midwifery and Social Care, Edinburgh Napier University, Edinburgh, UK; School of Health Sciences, University of Stirling, Stirling, UK

**Keywords:** Systematic review, Patient, Experience, Satisfaction, Quality, Hospital, Acute care, Instruments, Questionnaires, Surveys, Utility

## Abstract

**Background:**

Improving and sustaining the quality of hospital care is an international challenge. Patient experience data can be used to target improvement and research. However, the use of patient experience data has been hindered by confusion over multiple instruments (questionnaires) with unknown psychometric testing and utility.

**Methods:**

We conducted a systematic review and utility critique of questionnaires to measure patient experience of healthcare quality in hospitals. Databases (Medical Literature Analysis and Retrieval System (MEDLINE), Cumulative Index to Nursing and Allied Health Literature (CINAHL), Psychological Information (PsychINFO) and Web of Knowledge until end of November 2013) and grey literature were scrutinised. Inclusion criteria were applied to all records with a 10 % sample independently checked. Critique included (1) application of COSMIN checklists to assess the quality of each psychometric study, (2) critique of psychometric results of each study using Terwee et al. criteria and (3) development and critique of additional aspects of utility for each instrument. Two independent reviewers completed each critique. Synthesis included combining findings in a utility matrix.

**Results:**

We obtained 1157 records. Of these, 26 papers measuring patient experience of hospital quality of care were identified examining 11 international instruments. We found evidence of extensive theoretical/development work. The quality of methods and results was variable but mostly of a high standard. Additional aspects of utility found that (1) cost efficiency was mostly poor, due to the resource necessary to obtain reliable samples; (2) acceptability of most instruments was good and (3) educational impact was variable, with evidence on the ease of use, for approximately half of the questionnaires.

**Conclusions:**

Selecting the right patient experience instrument depends on a balanced consideration of aspects of utility, aided by the matrix. Data required for high stakes purposes requires a high degree of reliability and validity, while those used for quality improvement may tolerate lower levels of reliability in favour of other aspects of utility (educational impact, cost and acceptability).

**Systematic review registration:**

PROSPERO CRD42013006754

**Electronic supplementary material:**

The online version of this article (doi:10.1186/s13643-015-0089-0) contains supplementary material, which is available to authorized users.

## Background

Despite an array of improvement initiatives in hospitals, the quality of care delivered remains open to question [12, 14, 18]. Patients who have experienced hospitalisation can offer unique insights into quality of care, which can be used for improvement. Yet, patients’ views of quality of care are not always included in hospital measurement plans [4]. However, if patient experience data is to be used to improve quality of care in hospitals, it needs to be reliable and valid yet usable in practice [11, 54].

Measurement is fundamental to improving the quality of hospital care [43]. We will only know whether interventions or changes are working if processes and outcomes are measured. Measuring the patient experience in a robust way enables facts to be established from the complex phenomena of quality of care [32]. Patient experience data can be used to benchmark hospital performance, monitor effectiveness of interventions, establish hospital rankings and secure funding for research and innovation. Quantitative data can be combined with patient stories to create compelling evidence to evoke reflection and improvements within clinical teams [30]. Measuring the patient experience can highlight potential solutions, opportunities to improve hospital care.

Although a combination of tools is required to capture the complexity of hospital care, surveys are likely to remain the core method for measuring patient experience [11]. Surveys or questionnaires can be used to capture large samples of standardised data, which is essential if the patient perspective is to be equally represented alongside other aspects of care easier to quantify, such as waiting times.

There are, however, challenges to measuring the patient perspective of hospital care using questionnaires. Firstly, quality of care is difficult to quantify and define [5]. There is no widely accepted definition of quality of care; rather, there is an understanding that it is multi-dimensional, with varying interpretations dependent on who is being asked [16]. The widely accepted STEEEP acronym (Safety, Timeliness, Effectiveness, Efficiency, Equity and Person Centeredness) is most commonly used to describe the dimensions of quality of care [23]. There is consensus that quality of care consists of technical (knowledge and expertise) and interpersonal divisions (i.e. empathetic behaviour) [5, 16]. For example, the explanation of treatment options (technical) is improved if they are explained in an empathic and person-centred way (interpersonal).

Secondly, the terms ‘satisfaction’ and ‘experience’ are often used interchangeably despite their different meanings. Satisfaction is the gap between patient expectations and experience. Patients tend to overrate satisfaction, due to gratitude bias and other factors. Therefore, the validity and usefulness of satisfaction data is limited; thus, there are calls for the patients’ perspective of quality of care to focus on measuring experience, as opposed to satisfaction [31, 57, 58]. Patient experience is defined as things that happen to people and the extent that people’s needs are met [17]. Questions are, therefore, designed around what actually occurred during hospitalisation. For example, a question might be asked as to whether or not patients received the right medication, at the right time as opposed to asking patients to rate their satisfaction with medicine administration. The emphasis is on asking patients whether or not, or how often, they have experienced certain care processes, rather than on rating aspects of care or treatment.

Thirdly, instruments need to be valid and reliable. That is, they accurately represent the patient experience of hospital care (validity), and this is measured consistently (reliability). An example of validity would be ensuring the patient experience is being measured, rather than the clinicians’ perspective, as these are known to differ [16]. An unreliable tool would not be able to monitor improvement over time, consistently and without error.

Finally, instruments need to have high utility if they are to be used in real-world practice [3]. Van der Vleuten considered instrument utility from five aspects, namely validity, reliability, cost efficiency, acceptability and educational impact [52]. Each of these aspects is important to users of patient experience instruments. In the current financial climate, cost had become a key consideration when selecting an instrument. For example, obtaining a large, standardised sample will be expensive. Acceptability considers the suitability of the instrument from the users’ perspective. This includes not only measuring a valid construct but also the tolerability of the instrument. For example, users (patients, clinicians and managers) may think a questionnaire has an unacceptably high number of questions, despite internal consistency (reliability) being improved by increasing the number of items [10]. Educational impact is also a factor to consider. How easy is it for an organisation, or individual within it, to drill down and make use of the data? Van der Vleuten emphasises the importance of weighing all of these aspects to select the right instrument, for the right purpose. For example, if survey results are to be used for high stakes (the outcome has important consequences for an individual or organisation), there is a necessity for high reliability, while tolerating high cost. Data used for team improvement may tolerate lower levels of reliability but require educational impact and acceptability.

This systematic review critiques the utility of published questionnaires aiming to measure the adult inpatient experience of hospital quality of care. The findings will aid appropriate instrument selection, which will ultimately increase the likelihood of the patient’s voice improving hospital quality of care.

### Study objectives

1. Identify questionnaires available to measure the adult inpatient experience of general (medical/surgical) hospital quality care.
2. Identify studies conducted to examine the measurement properties (validity and reliability) of questionnaires quantifying the adult inpatient experience of quality care.
3. Identify papers exploring the cost efficiency, acceptability and educational impact of questionnaires measuring the adult inpatient experience of hospital quality care.
4. Critique the quality of the methods and results of the measurement properties using recognised criteria for each instrument.
5. Determine the utility of each questionnaire by integrating results on the quality of validity, reliability, cost efficiency, acceptability and educational impact.

## Methods

Our methods were published in a protocol [4] prior to conducting the review, and this study was registered with PROSPERO (registration number CRD42013006754). A PRISMA (2009) Checklist aided the study design (see Additional file 1).

### Search strategy

Search strategies were devised, and the following databases were searched from inception until end of November 2013 as follows: Medical Literature Analysis and Retrieval System (MEDLINE), Cumulative Index to Nursing and Allied Health Literature (CINAHL) and Psychological Information (PsychINFO). No restrictions were applied to language, publication type or year. The word ‘satisfaction’ was included in our strategies, as some papers pertaining to ‘experience’ were filed under satisfaction within Medical Index Subject Headings (MeSH) within databases. Other literature was identified by contacting experts in the field and searching specialist websites (see Additional file 2 for MEDLINE search strategy and resources searched). Some e-mails were not responded to; we set a definitive deadline for response for July 2014. All records were exported into Ref Works for removal of duplicates and reference management. Duplicate removal was second checked within Ref Works and amended by hand by MB.

### Selection criteria

An inclusion selection form was applied to all titles and abstracts, enabling a transparent and focused selection of papers of interest: [4]

*Study type*: examining any measurement properties, theoretical development or utility of a questionnaire.

*Population*: adult in-patients, thus excluding clinicians, family members and paediatric perspectives.

*Setting*: surgical or medical care, thus excluding specialist areas, such as palliative and psychiatric care as patients in specialist areas have different determinants of what constitutes quality of care [38, 44].

*Global perspective*: patients’ overall experience of hospital quality of care. Therefore, we eliminated condition-specific instruments and those measuring quality of specific professional groups.

*Construct of interest*: quality of care. We included all definitions or conceptualisations of quality, so long as they were defined from the patients’ perspective. Studies measuring patient satisfaction were eliminated due to the theoretical and methodological limitations identified earlier.

Where decisions could not be made on title or abstract alone, full papers were retrieved. A second reviewer independently applied the inclusion criteria to a random 10 % of the records, retrieving full papers where necessary.

### Data extraction/instrument overview

We used a data extraction form to standardise the information recorded and aid analyses [31]. Some instruments have been considered by multiple studies; therefore, papers were grouped according to the instrument type to reduce duplication of data extraction. Data was extracted from the most recent version of the instrument only. All data extracted were checked for accuracy by a second, independent researcher.

### Assessment of study quality

The Consensus-based Standards for the Selection of Health Measurement Instruments (COSMIN) checklist was used to evaluate the methodological rigour of the studies [34, 51], and Quality Criteria for Measurement Properties [50] was used to critique the results of the studies. Studies were not rejected on the basis of this quality critique; rather, the results were synthesised to enable appropriate instrument selection.

The COSMIN checklists have been designed and validated for use in evaluating the rigour of psychometric studies of healthcare instruments [34]. The COSMIN checklist provides separate checklists (referred to as boxes) for each type of measurement property, for example, box A is for internal consistency, B for reliability and so forth. Boxes A–H are for different types of psychometric studies and have their own associated quality questions. See Mokkink et al [34] for a full explanation of the COSMIN checklist. The checklists for interpretability and generalisability were not used as these are recommended for data extraction use only and are not scored for quality. All quality grading of studies were scored independently by two researchers (MB, DM) before reaching consensus.

**Fig. 1:**
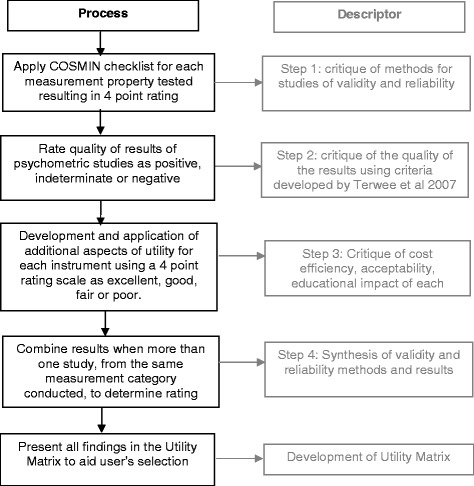
Quality critique procedure

There were several steps in the quality critique of retained studies and instruments (Fig. 1 of quality critique procedure). Firstly, we applied the appropriate A–H checklist to critique the methodological quality of how each measurement property was being tested within each study. Responses within individual checklists were given a methodological score by applying the COSMIN four-point checklist scoring system. The scoring system is designed to ensure that items are scored as ‘excellent’ when there is evidence of adequate methodological quality, ‘good’ when relevant information is not fully reported but adequate quality can be assumed, ‘fair’ if the methodological quality is in doubt and ‘poor’ when there is evidence that the methodological quality is not adequate. Where answers to checklist questions were of variable ratings (i.e. some excellent, some poor), the overall score was determined by taking the lowest rating of any item. In other words, the worst score counted [51].

Secondly, we rated the quality of the results of the psychometric studies by using the Quality Criteria for Measurement Properties devised by Terwee et al. (see Table 1) [50]. Results were rated as positive (+), indeterminate (?) or negative (−) according to the quality criteria for each measurement property. For example, positive ratings for internal consistency are given, using Terwee et al. criteria, if Cronbach’s alpha is ≥0.70. Studies with Cronbach’s alpha results of <0.70 would be categorised as negative, or where Cronbach’s alpha was not determined, the result would be categorised as indeterminate. A full explanation, with justification for all COSMIN criteria results, is available from Terwee et al. [50].

**Table 1:** Quality Criteria for Measurement Properties (Terwee et al. 2007) [50]

Property	Rating	Quality criteria
Reliability		
Internal consistency	(+)	(Sub)scale unidimensional AND Cronbach’s alpha(s) ≥0.70
	?	Dimensionality not known OR Cronbach’s alpha not determined
	(−)	(Sub)scale not unidimensional OR Cronbach’s alpha(s) <0.70
Measurement error	(+)	MIC > SDC OR MIC outside the LOA
	?	MIC not defined
	(−)	MIC ≤ SDC OR MIC equals or inside LOA
Reliability	(+)	ICC/weighted Kappa ≥0.70 OR Pearson’s *r* ≥ 0.80
	?	Neither ICC/weighted Kappa, nor Pearson’s *r* determined
	(−)	ICC/weighted Kappa <0.70 OR Pearson’s *r* < 0.80
Validity		
Content validity	(+)	The target population considers all items in the questionnaire to be relevant AND considers the questionnaire to be complete
	?	No target population involvement
	(−)	The target population considers all items in the questionnaire to be irrelevant OR considers the questionnaire to be incomplete
Construct validity		
Structural validity	(+)	Factors should explain at least 50 % of the variance
	?	Explained variance not mentioned
	(−)	Factors explain <50 % of the variance
Hypothesis testing	(+)	Correlation with an instrument measuring the same construct ≥50 % OR atleast 75 % of the results is in accordance with the hypotheses) AND correlation with related constructs is higher than with unrelated constructs
	?	Solely correlations determined with unrelated constructs
	(−)	Correlation with an instrument measuring the same construct <50 % OR <75 % of the results is in accordance with the hypotheses OR correlation with related constructs is lower than with unrelated constructs

*+* positive, *−* negative, *?* indeterminate, *AUC* area under the curve, *MIC* minimal important change, *ICC* intraclass correlation, *SDC* smallest detectable change, *LOA* limits of agreement

### Development of quality matrix

The COSMIN checklists only enable a critique of the validity and reliability aspects of utility; as a third step in devising a quality matrix, we developed additional questions to rate the cost efficiency, acceptability and educational impact of instruments (Table 2). Each question response has a four-point rating criteria of excellent, good, fair or poor.

**Table 2:** Additional aspects of utility scoring criteria

	Excellent (****)	Good (***)	Fair (**)	Poor (*)
Questions for cost efficiency				
1. What are the number of observations (patients, raters, times) needed to reach the required level of reliability for the purpose of the instrument?	Only a small sample needed (<30)	A moderate sample size (30–49)	Not explicit but can be assumed or (50–99 assessments needed)	No details given or (≥100 assessments needed)
2. How long does an assessment take to complete	≤15 min	≤ 30 min	30–60 min	>60 min
3. What are the administrative costs of completing the assessment?	Easily embedded within existing resource. Little additional support required	Some administrative resource but no specialist resource required	Large amount of resource required to assess and administer	Significant specialist expertise and administrative time required to assess and administer
4. What is the cost to complete a reliable sample?	Minimal	Moderate	Considerable	Extensive
Questions for acceptability				
1. Is there evidence of subjects understanding of the instrument/assessment?	Investigations of subjects understanding (i.e. cognitive testing of instruments)	Estimated evidence of subjects understanding (i.e. high number of questions missed)	Subject understanding not explicitly stated but some can be assumed (i.e. student guide to OSCE)	No evidence of subject understanding
2. How many assessments are not completed?	There are low numbers of missing items (<10 %) and adequate response rates (>40 %)	There are a high number of missing items (>10 %) and an adequate response rates (>40 %)	There are low numbers of missing items or poor (<10 %) and an inadequate response rate (<40 %)	There are high numbers of missing items (>10 %) and poor response rates (<40 %)
3. Has the instrument/assessment been tested in an appropriate context?	Evidence of successful administration/use within an appropriate setting	Tested in vivo and changes recommended would be achievable	Testing in vivo and changes recommended would be difficult or only partially tested in vivo	Testing has only been conducted in vitro/simulation
Questions for educational impact				
1. There is evidence of the instruments intended purpose being achieved (i.e. if aim is to enable hospital ranking for patient selection, is there evidence that the results are actually influencing patient choice?)	Clear evidence of intended purpose being fulfilled	Explanatory or theoretical link between intended and actual use but no clear evidence	Evidence of theoretical work but relationship between intended and actual purpose poorly or not described	No evidence of intended purpose becoming actual
2. The scoring system is easily translated or available in an easy to use format?	Explicitly stated and easy to calculate	Explicitly stated but not easy to calculate	Scoring only calculated by resource with statistical knowledge	Scoring not explained well enough to calculate
3. The feedback from the results can be readily used for action where necessary?	Feedback is readily available in a format that enables necessary action	Feedback is readily available but not drilled down enough to enable targeted action	Minimal feedback available or delay results in limited impact	No explanation to determine adequacy of feedback. No direct feedback could be readily used without additional expertise

Cost efficiency was rated in terms of the resources necessary to utilise the instrument for its primary purpose. The higher the resource/cost required, the lower the rating. Sample sizes detailed in instrument papers were used to answer the first question ‘What are the number of observations (patients, raters, times) needed to reach the required level of reliability for the purpose of the instrument?’ The number of observations needed to achieve the desired level of reliability is important to establish in terms of feasibility [35]. An instrument may be highly reliable but require extensive resource to obtain a reliable sample. Therefore, we are determining the resources necessary to achieve the level of reliability necessary for the instrument’s primary purpose. For example, the Hospital Consumer Assessment of Healthcare Providers and Systems (HCAHPS) instrument requires a minimum of 300 questionnaires per hospital to achieve a minimum of 0.8 reliability for all reported measures [20]. Also, if an instrument requirement was use on two or more occasions to obtain reliability (i.e. test re-test reliability) where time affected the instrument performance, there would be a need to multiply the number of assessments by the given number of administrations.

Another question estimated the resource required to administer the questionnaire, for example, assessments requiring to be conducted by experts are more expensive in comparison to self-completion questionnaires. Completion time was also included; where developers had not published information on completion times, estimates were calculated by comparing with similar instruments. Question 4 brought together the preceding three questions on cost efficiency to estimate the cost of obtaining a reliable sample: minimal, moderate, considerable or extensive. These categories transformed into an inverse rating scale from poor to excellent, ‘extensive’, for example, becoming a rating of ‘poor’ for cost efficiency.

For the utility dimension of acceptability, questions were designed around evidence of the subjects’ perception of the instrument, where less acceptance would result in a lower rating. There is an overlap between this category and content validity. However, the COSMIN checklist for content validity does not cover all aspects of user acceptability, e.g. cognitive testing. Also, some instruments may demonstrate content validity but have only been tested in a simulated environment or have an unacceptably high number of questions. Grading was determined on a four-point rating scale of excellent, good, fair and poor. The overall rating of acceptability was determined by the worst score.

Questions for educational impact required evidence around an instrument’s ease of use for learning or decision-making. Using a validated and reliable instrument is futile if not followed by action, learning or impact. This category determines how easy it is to make use of the instrument results as intended. Again, question responses were graded using four rating responses, with the final rating determined by the worst score.

Where responses within individual categories of utility dimensions differed, the overall score was determined by the worst score counts, except for cost efficiency, where scoring was based on a balance of responses. Questions and categorised responses were refined following the testing of application to one instrument. Two researchers independently scored all papers and resolved disagreements through consensus.

#### Beattie and Murphy instrument utility matrix

All results were integrated into a utility matrix to aid instrument selection for users. The matrix enabled a synthesis of the quality of the methods used in the studies and results of all measurement properties from each study of each instrument, from the application of COSMIN and Terwee et al. criteria [50]. To simplify, the results from validity studies were merged into three headings: content, construct and criterion validity. Content validity included any study on the theoretical development of the instrument construction. Studies empirically testing any other type of validity, except criterion, were grouped together as construct validity. Construct validity is an overarching term for validity as opposed to a distinct form [10]. However, criterion validity was retained as a separate category as this is viewed as the ‘gold standard’, indicating the ability of an instrument to predict future outcomes, which would be of interest to those selecting an instrument.

Reliability was presented in the matrix in two categories: internal consistency and other forms of reliability. Internal consistency is the relationship between items and accounts for error generated by the questions or items asked by the instrument [49]. Measurement of internal consistency is only relevant when instruments have been designed from a reflective model. To determine whether instruments derived from a reflective model, we asked the question ‘Do we expect all items to change when the construct changes?’ If changes to the patient experience of quality of care did not result in changes in all domains, we classified the questionnaire as derived from a formative model. Also, measures of internal consistency are based on a single administration of the instrument and essentially represent the average of correlations among all the items in the instrument [49]. However, this does not account for the potential error between different observers or from one time interval to another. Generalizability G-theory and its associated decision D-studies can be used to further explore the reliability of an instrument and research the most effective blend of relevant resources (times of administration, number of observers or raters) needed to explain error and attain reliability [20, 49]. To address the potential for misinterpreting an instrument as reliable when demonstrating high internal consistency but where other sources of error had not been examined, we added a question to the matrix to indicate whether or not all relevant sources of errors were investigated.

We presented ratings of study quality in star ratings: excellent (****), good (***), fair (**) and poor * and the quality of results as positive (+), (?). Where more than one study from the same measurement category had been conducted, we determined the average point to rate the quality of the study methods. We provide two examples of combining validity and reliability scores to further explain. Example 1: if structural validity scored ‘excellent’ and cross-cultural validity scored ‘fair’, our overall rating would be ‘good’. If, however, structural validity scored ‘excellent’ and cross-cultural validity scored ‘good’, we would rate validity overall as good to excellent (represented as ***/****). Example 2: if the same instrument had two studies on reliability with study quality for one scoring ‘excellent’ and the other scoring ‘good’, we would rate reliability overall as good to excellent (represented as ***/****). Where the quality of study results varied, within the same measurement property, we presented these as mixed. For example, if structural validity results scored positive and cross-cultural validity scored negative, we presented these as mixed (+/−).

## Results

**Fig. 2:**
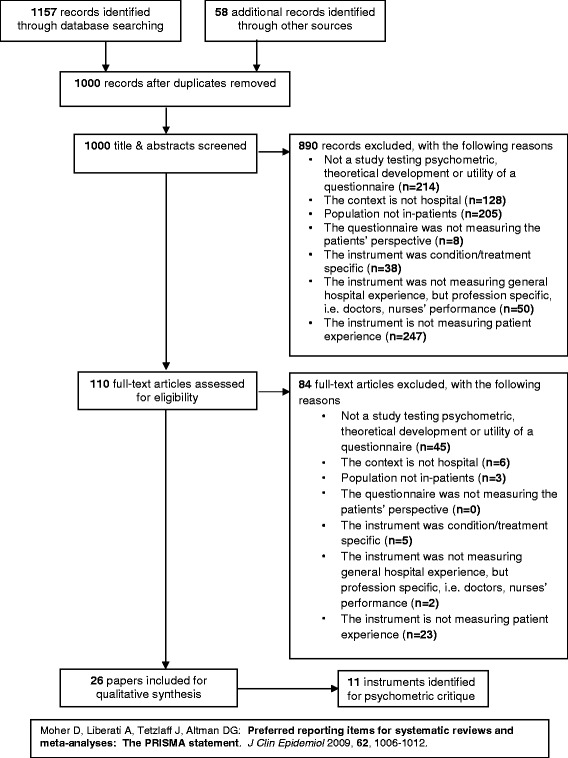
Modified PRISMA flow diagram

Results of the search strategy were documented within the PRISMA flow diagram (see Fig. 2) [33]. We obtained 1157 records from our searches. Following removal of duplicates, 1000 records were screened for inclusion criteria. Application of the inclusion criteria to titles and abstracts resulted in the exclusion of 890 records. We retrieved 110 full-text articles where we were unable to make decisions from the title and abstract. Following application of inclusion criteria to full-text articles, we rejected 84 and retained 26 papers.

### Screening results

A second reviewer applied the inclusion criteria to a random 10 % of the 1000 papers (*n* = 100). Where the second reviewer was unable to make a decision on title and abstract alone, full-text papers were retrieved (*n* = 17). We rejected numerous papers where the outcome of interest, or theoretical model, was patient satisfaction, as opposed to patient experience (see Fig. 2 for specific exclusion results). The percentage of agreement between both reviewers was 90 %, therefore demonstrating a highly reliable process. Reviewers reached consensus following discussion on the remaining ten papers. The process resulted in 26 papers being retained in relation to 11 instruments measuring the patient experience of hospital quality of care.

### Characteristics of included instruments

The range of instruments and associated papers can be found in Table 3. Instruments were available across the World: Ethiopia (1), Hong Kong (1), India (1), Scandinavia (4), UK (3) and USA (1). Most instruments had generated multiple versions as they developed over time; therefore, we critiqued the most recent instrument version and associated psychometric studies published in November 2013. For example, we used the Scottish Inpatient Patient Experience Survey (SIPES) measure version 2012 [46] as there is approximately a 1-year time lag between the instrument’s use and results. Some instruments had extensive developmental histories, for example, the National Health Service Inpatient (NHSIP) Survey has been operating annually since 2002 [40], but its theoretical development work can be traced back to as early as 1991, to the original Picker Adult In-Patient survey [2, 9, 19]. We included the most recent works only. The Hospital Consumer Assessment of Healthcare Providers and Systems (HCAHPS) originated in 2002 [1], but we used version 2012 [2].

**Table 3:** Instrument overview

Instrument/abbreviation	Associated papers	Country of origin	Domains covered	Conceptual framework	No. of items	Mode of administration	Timing of administration
Hospital Consumer Assessment of Healthcare Providers and Systems (HCAHPS)	Sofaer et al. [48]	USA	Nurse communication	Reflective	24	Mail	48 h—6 weeks of discharge
	Keller et al. [26]		Doctor communication			Telephone	
	O’Malley [36]		Physical comfort			Mail with telephone follow-up	
	Levine et al. [29]		Pain control				
	Giordano et al. [20]		Medicine communication				
	Agency for Healthcare Research and Quality [1]		Discharge information			Interactive Voice Recognition (IVR)	
	Centers for Medicare and Medicaid [8]		Responsiveness to patient				
Quality from the Patients' Perspective (QPP)	Wilde et al. [56]	Sweden	Medical-technical competence	Reflective	68	Self-completion questionnaire	At discharge
	Wilde et al. [55]		Physical technical conditions				
	Larsson et al. [28]		Personal necessities				
			Characteristics				
			Identity-orientated approach				
			Situation				
			Participation				
			Commitment				
			Socio-cultural atmosphere				
			Positive treatment of significant others				
Quality from the Patients' Perspective Shortened (QPPS)	Larsson et al. [27]	Sweden	Medical-technical competence	Reflective	24	Self-completion questionnaire	At discharge
			Physical technical conditions				
			Identity-orientated approach				
			Socio-cultural atmosphere				
Picker Patient Experience Questionnaire (PPE-15)	Jenkinson et al. [25]	England	Information and education	Reflective	15	Self-completion postal questionnaire	Within 1 month of discharge
	Jenkinson et al. [24]		Coordination of care				
	Reeves et al. [42]		Physical comfort				
			Emotional support				
			Respect for patient preferences				
			Involvement of family and friends				
			Continuity and transition				
			Overall impression				
NHS Inpatient Survey (NHSIP)	Boyd [6]	England (originated in the USA)	Admission to hospital	Formative	70	Postal survey	Between 4 and 5 months of discharge
	Sizmur and Redding [47]		The hospital and ward				
	Picker Institute Europe [40]		Doctors				
	Decourcy et al. [13]		Nurses				
			Your care and treatment				
			Operations and procedures				
			Leaving hospital				
Scottish Inpatient Patient Experience Survey (SIPES)	Scottish Government [46]	Scotland	Admission to hospital	Formative	30	Postal survey, questionnaire (also available online, by telephone and via text phone)	Between 4 and 5 months of discharge
			The hospital and ward				
	Scottish Government [45]						
			Care and treatment				
			Hospital staff				
			Arrangements for leaving hospital				
			Care and support services after leaving hospital				
Hong Kong Inpatient Experience Questionnaire (HKIEQ)	Hospital Authority [22]	Hong Kong	Prompt access	Reflective	62	Mixed	48 h—1 month after discharge
			Information provision			92 % interviewed by telephone	
	Wong et al. [59]						
			Care and involvement in decision-making				
			Physical and emotional needs			8 % face-to-face home interviews	
			Coordination of care				
			Respect and privacy				
			Environment and facilities				
			Handling patient feedback				
			Overall care of health professionals and quality of care				
Patient Experience Questionnaire (PEQ)	Pettersen et al. [39]	Norway	Information future complaints	Reflective	35	Postal self-completion questionnaire	6 weeks after discharge
			Nursing services				
			Communication				
			Information examinations				
			Contact with next-of-kin				
			Doctor services				
			Hospital and equipment				
			Information medication				
			Organisation				
			General satisfaction				
Norwegian Patient Experience Questionnaire (NORPEQ)	Oltedal [37]	Norway	Whether doctors were understandable	Reflective	8	Self-completion	Within 7 weeks of discharge
			Doctors professional skills				
			Nurses professional skills				
			Nursing care				
			Whether doctors and nurses were interested in the patients problems				
			Information on tests				
Patient Experiences with Inpatient Care (I-PAHC)	Webster et al. [53]	Ethiopia	Nurse communication	Reflective	16	Interviewer-assisted completion	After first day of admission
			Doctor communication				
			Physical environment				
			Pain management				
			Medication and symptom communication				
Patient Perceptions of Quality (PPQ)	Rao et al. [41]	India	Medicine availability	Reflective	16	Interviewer-assisted completion	Not specified
			Medical information				
			Staff behaviour				
			Doctor behaviour				
			Hospital infrastructure				

Instruments covered similar domains to capture the patient experience of their hospital care. Some focused on stages of the patient’s journey, from admission to hospital discharge [6, 46]. Others were structured around dimensions of hospital quality, i.e. communication and coordination of care, such as HCAHPS [2] and Quality from the Patients’ Perspective Shortened (QPPS) [56]. All instruments covered aspects of technical and interpersonal components of quality of care. There were some cultural differences in content. For example, the Patient Perceptions of Quality (PPQ) [41] included questions around medicine availability, reflective of the low-income context in which the instrument was tested. Importantly, all instruments were measuring the patient experience, as opposed to satisfaction.

Most instruments were devised from a reflective model (see Table 3). That is to say, collectively, factors within the questionnaire reflect the construct of interest, patient experience of hospital quality. For example, changes made to improve the quality of hospital care (construct) would likely cause variation in all indicators, i.e. safety and person centeredness within these instruments. The NHSIP and SIPES instruments were exceptions, based on a formative model. Domains within their questionnaire were designed around the patient journey, i.e. from admission to discharge home. A poor experience during admission to hospital (indicator) would decrease the patient’s score of quality of care, but not necessarily influence other indicators, i.e. the patient’s experience of hospital discharge.

The number of items within the instruments varied from 8 to 70, excluding demographic questions. All instruments were self-completed instruments, except Patient Experiences with Inpatient Care (I-PAHC) and PPQ which required interviewer assistance due to the prevalence of illiteracy in the countries in which they were tested [41, 53]. Most instruments were mailed, although some offered telephone assistance (HCAHPS, SIPES, NHSIP) and HCAHPS was available in several formats (mail only, telephone only, mail followed by telephone and interactive voice response) [8].

All instruments were administered following discharge from hospital, except I-PAHC which was completed any time during the admission, but after the first day of hospitalisation [53]. Timings varied, from instruments being distributed on discharge to several months following hospitalisation.

### Instrument quality and results

The type and quality of the methods and results of the psychometric studies was variable but mostly of a high standard (see Table 4). Every instrument had evidence of examining at least one aspect of validity and reliability.

**Table 4:** Quality of methods and results of psychometric studies

Instrument/abbreviation	Associated papers	Measurement property	Result	Quality rating of results	Quality rating of methods
Hospital Consumer Assessment of Healthcare Providers and Systems (HCAHPS)	Sofaer et al. [48]	Content validity	Patients considered other aspects of hospital care which appear to have not been included	Negative	Poor
	Keller et al. [26]	Internal consistency	Cronbach’s alpha 0.70	Positive	Excellent
	Keller et al. [26]	Reliability	ICC 0.70	Positive	Excellent
	Keller et al. [26]	Structural validity	7 categorises for 16 items. Factor loadings 0.57–91. Uniqueness of error reported	Indeterminate	Excellent
	O’Malley [36]	Measurement error	Correlation between same composites different services	Indeterminate	Good
			Surgery 0.76		
			Obstetrics 0.73		
			Medical 0.85		
Quality from the Patients' Perspective (QPP)	Wilde et al. [56]	Content validity	35 patient interviews—development of relevant questionnaire	Positive	Excellent
	Wilde et al. [55]	Internal consistency	Cronbach’s alpha 0.80	Positive	Excellent
	Wilde et al. [55]	Content validity	High patient ratings of item clarity and comprehensiveness	Positive	Excellent
	Wilde et al. [55]	Structural validity	Factor solutions	Positive	Good
			Medical/technical competence 50.4 %		
			Physical/technical conditions 44.8 %		
			Identity-orientated approach 66.9 %		
			Socio-cultural atmosphere 65.8 %		
	Wilde et al. [55]	Criterion validity	Correlation between long and short version in their entirety was 0.90	Positive	Poor
	Larsson et al. [28]	Structural validity	RMSEA of 0.050 was obtained indicating the model was an acceptable fit	Indeterminate	Good
Quality from the Patients' Perspective Shortened (QPPS)	Larsson et al. [27]	Internal consistency	Cronbach’s alpha 0.74 for overall scale	Positive	Excellent
	Larsson et al. [27]	Criterion validity	Pearson correlation coefficients all results statistically significant 0.0025 when Bonferroni corrections made	Positive	Excellent
Picker Patient Experience Questionnaire(PPE-15)	Jenkinson et al. [25]	Internal consistency	Cronbach’s alpha 0.8	Positive	Good
	Jenkinson et al. [24]	Internal consistency	0.89 for 4 pages	Positive	Excellent
			0.87 for 12 pages		
	Reeves et al. [42]	Content validity	Focus groups, cognitive testing, amendments—research did not identify any missing items from patients’ perspective	Positive	Excellent
	Jenkinson et al. [25]	Criterion validity	Correlations between short and long version between 0.93 (*P* < 0.001) and 0.95 (*P* < 0.001)	Positive	Good
	Jenkinson et al. [24]	Hypothesis testing	Item correlations were above recommended levels for all PPE items in both survey versions (0.37–0.61)	Positive	Excellent
NHS Inpatient Survey (NHSIP)	Boyd [6]	Content validity	Tested and modified with group of inpatients	Positive	Excellent
	Sizmur and Redding [47]	Internal consistency	Item correlations given but Cronbach’s alpha not reported	Indeterminate	Fair
Scottish Inpatient Patient Experience Survey (SIPES)	Scottish Government [45]	Content validity	Extensive work with patient groups: survey, focus groups, stakeholder consultations, cognitive testing. Findings, the patient found the items relevant and comprehensive	Positive	Excellent
	Scottish Government [45]	Internal consistency	Cronbach’s alpha over 0.70 for each survey section	Positive	Poor
Hong Kong Inpatient Experience Questionnaire (HKIEQ)	Hospital Authority [22]	Internal consistency	Cronbach’s alpha 0.75 for overall scale	Positive	Fair
	Hospital Authority [22]	Reliability	Intraclass correlation 0.42–0.96 and test re-test 0.78	Positive	Fair
	Hospital Authority [22]	Content validity	Participants found the questionnaire to be clear, understandable, and appropriate	Positive	Excellent
	Hospital Authority [22]	Structural validity	17 factors explained 74 % of the variance	Positive	Fair
	Wong et al. [59]	Internal consistency	Cronbach’s alpha 0.75 for overall scale	Positive	Fair
	Wong et al. [59]	Structural validity	18 factors explained 75.5 % of the variance	Positive	Fair
	Hospital Authority [22]	Cross-cultural validity	Translated but not cross-culturally validated	Indeterminate	Fair
Patient Experience Questionnaire (PEQ)	Pettersen et al. [39]	Internal consistency	Cronbach’s alpha greater than 0.70 for overall scale	Positive	Fair
	Pettersen et al. [39]	Reliability	Test re-test 0.62–0.85 with ICC exceeding 0.7	Positive	Fair
	Pettersen et al. [39]	Content validity	Grouped more than 600 m written comments and held focus groups with previous inpatients to ensure relevant and sufficient items were covered	Positive	Good
	Pettersen et al. [39]	Structural validity	20 items, 6 factors accounted for 67 % total variance	Positive	Excellent
	Pettersen et al. [39]	Hypothesis testing	Associations between rating scale and external measures, i.e. gender, age, fulfilment of expectations. Only mean differences computed	Indeterminate	Poor
Norwegian Patient Experience Questionnaire (NORPEQ)	Oltedal [37]	Internal consistency	Item correlation 0.59–0.71 and Cronbach’s alpha 0.85	Positive	Fair
	Oltedal [37]	Reliability	Intraclass correlation 0.45–0.79 and test re-test 0.88	Positive	Good
	Oltedal [37]	Content validity	Patient interviews found questions and scaling easy to understand and all relevant questions covered	Positive	Good
	Oltedal [37]	Structural validity	6 items explained 57.7 % variance	Positive	Good
	Oltedal [37]	Construct validity	Hypothesised scales scores would correlate 0.6–0.8 with satisfaction (correlation significant, range from high to low)	Positive	Good
			Scale scores would correlate 0.4–0.6 perceptions of incorrect treatment (moderate result)		
			Scores would correlate 0.1–0.3 with patient health and physical health. (Result 0.19–0.27)		
Patient Experiences with Inpatient Care (I-PAHC)	Webster et al. [53]	Internal consistency	Cronbach’s alpha >0.78	Positive	Excellent
	Webster et al. [53]	Content validity	Focus groups, revisions by stakeholders, translated, cognitively tested and patient groups reported clear questions covering all aspects important to them	Positive	Excellent
	Webster et al. [53]	Structural validity	Kept if item loadings greater than 0.40. Variance not reported	Indeterminate	Excellent
	Webster et al. [53]	Construct validity	5 factors with loadings 0.48–0.86. Results in accordance with priori hypothesis	Positive	Excellent
	Webster et al. [53]	Cross-cultural validity	Translation done but not empirically tested	Indeterminate	Fair
Patient Perceptions of Quality (PPQ)	Rao et al. [41]	Internal consistency	Cronbach’s alpha >0.70	Positive	Excellent
	Rao et al. [41]	Content validity	Questionnaire devised from qualitative interviews with patients	Positive	Excellent
	Rao et al. [41]	Structural validity	5 dimensions explained 73 % variance	Positive	Excellent

### Validity

Content validity was tested for all instruments by exploring which aspects of hospital quality care mattered most to patients. Scores for content validity were rated as good or excellent, except for HCAHPS [48]. HCAHPS was rated as poor as no information was provided to determine whether aspects of quality suggested by patients had been integrated within their instrument, as well as patients having concurred with pre-determined items. While the quality of the methodology and results was limited for HCAHPS, in all other instruments, the questionnaire items were relevant and sufficient, therefore rating positive for content validity.

All instruments had examined other types of validity, except NHSIP and SIPES. Comments in NHSIP documentation referred to previous structural validity, but the detail required to judge criteria was unavailable [47]. Criterion validity is considered when an instrument is compared with a gold standard. While no gold standard exists for measures of patient experience, the COSMIN criteria include comparisons of shortened with original longer versions as criterion validity. Three studies comparing shortened versions with their original longer versions (QPP [55], QPPS [27], PPE-15 [24, 25]), rated fair, excellent and good, respectively, with positive results. Some developers had tested the validity of their instrument extensively, namely QPP, HKIEQ and NORPEQ which had conducted three or more validity studies. The methodological quality of all construct validity studies was mostly good or excellent (HCAHPS), except HKIEQ. [22] HKIEQ was rated as fair as no description was given on how the authors handled missing items within their study. Most results of construct validity were categorised as positive, as factor analysis explained at least 50 % of the variance or met other Quality Criteria for Measurement Properties identified by Terwee et al. (see Table 1) [50]. Several studies were rated as indeterminate as they did not meet the Quality Criteria for Measurement Properties’ results. For example, structural validity was thoroughly examined for the HCAHPS instrument but was categorised as indeterminate as structural equation modelling does not report factor loadings [26]. This result needs to be interpreted with caution as the HCAHPS study demonstrated an excellent fit for structural validity. The methodological quality of criterion validity for the QPP instrument was rated as poor as there were flaws identified in the study design [55]. The validity of one QPP study [55] was in doubt as student nurses were given scenarios to act as simulated patients to answer questionnaire items in the instrument.

### Reliability

All instruments studied internal consistency to determine the interrelatedness among items. All instruments achieved positive internal consistency results, except NHSIP [47] which was indiscriminate as Cronbach’s alpha was not determined. Importantly, two instruments [45, 47]. were derived from formative models and did not have unidimensional subscales, which is reflected in their indiscriminate results and lower quality findings [25, 47]. However, the quality of the study methods for five instruments (NHSIP [47], SIPES [45], HKIEQ [22, 59], PEQ [39] and NORPEQ [37]) did not clarify how missing items were handled. Four instruments examined types of reliability in addition to internal consistency (HCAHPS [26], HKIEQ [22], PEQ [39] and NORPEQ [37]). All had positive results, but one HCAHPS study was indeterminate as the minimal important change was not determined as per the Quality Criteria for Measurement Properties (as detailed in Table 1).

### Results of instrument utility

The cost efficiency was rated as good for QPPS [27], NORPEQ [37] and I-PAHC [53]. All other instruments were rated as poor or fair, highlighting that considerable or extensive resource would be required to obtain an adequate sample (see Table 5). All instruments, except QPP, were rated excellent or good for the dimension of acceptability, as there was evidence of user acceptability in an appropriate context. QPP was rated as fair due to the evidence of testing in a simulated setting only [56].

**Table 5:** Results of additional aspects of utility

	HCAHPS		QPP		QPPS		PPE-15		NHSIP		SIPE		HKIEQ		PEQ		NORPEQ		I-PAHC		PPQ	
	F	R	F	R	F	R	F	R	F	R	F	R	F	R	F	R	F	R	F	R	F	R
**Cost efficiency**																						
1. What are the number of observations (patients, raters, times) needed to reach the required level of reliability for the purpose of the instrument?	≥300 [20]	Poor	Not reported	Poor	Not reported	Poor	330 per group [24]	Poor	Not Reported	Poor	Variable but >100	Poor	300–500 [45]	Poor	Not specified	Poor	Not specified	Poor	≥230 [53]	Poor	Not specified	Poor
2. How long does an assessment take to complete?	8 min [8]	Good	30 min [28]	Good	≤15 min	Excellent	12 min [42]	Excellent	20 min (estimate)	Good	20 min [46]	Good	25 min [59]	Good	<30 min (estimate)	Good	>15 min (estimate)	Excellent	15 min [53]	Excellent	<30 min (estimate)	Good
3. What are the administrative costs of completing the assessment?	V large numbers and expertise [8]	Poor	Considerable [28]	Fair	Brief and easy scoring [27]	Excellent	Large no. and standardised data	Fair	Large no. and standardised	Fair	V large numbers and expertise	Poor	V large numbers and expertise	Poor	Considerable	Fair	Brief and simple scoring	Good	Interviewers required	Fair	Interviewer required [41]	Fair
4. What is the cost to complete a reliable sample?	Extensive	Poor	Considerable	Fair	Minimal	Good	Considerable	Fair	Extensive	Poor	Extensive	Poor	Extensive	Poor	Considerable	Fair	Moderate	Good	Moderate	Good	Considerable	Fair
Overall Rating	POOR		FAIR		GOOD		FAIR		POOR		POOR		POOR		FAIR		GOOD		GOOD		FAIR	
**Acceptability**																						
1. Is there evidence of subjects understanding of the instrument/assessment?	Yes [29,48]	Excellent	Yes [55]	Excellent	Yes [8]	Excellent	Yes [42]	Excellent	Yes [47]	Excellent	Yes [45]	Excellent	Yes [22]	Excellent	Yes [39]	Excellent	Yes [37]	Excellent	Yes [53]	Excellent	Yes [41]	Excellent
2. How many assessments are not completed?	25 % miss RR 47 %	Good	13 % miss RR 68 % [55]	Good	25 % miss RR 79 % [55]	Good	29 % miss RR 68 % [42]	Good	No info RR 49 % [47]	Good	No info RR 50 % [13]	Good	21 % miss RR 49 % [22]	Good	>10 % mis RR 53 % [39]	Excellent	42.5 %mis RR 48 % [37] 85 %	Excellent	High No RR 95 % [53]	Good	0 % miss RR 85 % [41]	Excellent
3. Has the instrument/assessment been tested in an appropriate context?	Yes [26]	Excellent	Tested in simulation [55]	Fair	Yes [55]	Good	Yes [42]	Excellent	Yes [47]	Excellent	Yes [45]	Excellent	Yes [22]	Excellent	Yes [39]	Excellent	Yes	Excellent	Yes	Excellent	Yes	Excellent
Overall Rating	Good		Fair		Good		Good		Good		Good		Good		Excellent		Excellent		Good		Excellent	
**Educational impact**																						
1. Is there evidence of the instrument being used for its intended purpose? (i.e. if aim is to provide hospital ranking for patient selection, is there evidence that the results are influencing patient choice?)	Evidence of purpose [20]	Excellent	Discussion of purpose but no evidence [55]	Fair	Discussion of purpose but no evidence [27]	Fair	Explanatory use for national comparison	Good	Clear evidence of purpose [47]	Excellent	Explanatory use for national comparison [45]	Good	Explanatory use for national benchmarking [22]	Good	Clear evidence of purpose [39]	Excellent	Explanatory use described [37]	Good	Explanatory use described [53]	Good	Explanatory use described [41]	Good
2. Is the scoring system easily translated or available in an easy to use format?	Easy scoring	Excellent	Easy scoring	Excellent	Easy scoring	Excellent	Easily scored	Excellent	Statistical knowledge	Fair	Easy colour coding	Excellent	Statistical expertise	Fair	Not explained	Poor	Easy scoring	Excellent	Easy scoring	Excellent	Easy scoring	Excellent
3. Can the results be readily used for action where necessary?	Available but not at unit/team level	Good	Results actionable at local level	Excellent	Results actionable at local level	Excellent	Adjustments needed (Jenkinson comparison)	Fair	Expertise required to enable local action	Fair	Results at hospital level	Good	Results at hospital level	Good	No information	Poor	Readily available	Excellent	Readily available	Excellent	Readily available	Excellent
Overall Rating	Good		Fair		Fair		Fair		Fair		Good		Fair		Poor		Good		Good		Good	

*F* findings, *R* ratings

Educational impact was good for five of the instruments (HCAHPS [26, 29, 48], SIPES [45, 46], NORPEQ [37], I-PAHC [37], PPQ [53]) as there was evidence of the instruments being easily used for their intended purpose, i.e. hospital ranking or quality improvement. Five instruments (QPP [55], QPPS [27], PPE-15 [25], NHSIP [13, 40], HKIEQ [22]) were rated as fair as there was some evidence of educational impact, and PEQ was rated as poor as there was no enough information to determine educational impact.

### Utility matrix results

All results (critique of methods, results and additional aspects of utility) were embedded in our utility matrix to enable an easy overview and aid instrument selection (see Table 6). We found two main purposes of patient experience instrument use to compare performance across hospitals and local quality improvement. Overall, HCAHPS, NORPEQ, PPE-15 and I-PAHC demonstrated the most evidence that their instruments were valid and reliable. NHSIP and SIPES demonstrated the least evidence of validity and reliability. All other instruments were found to have a degree of psychometric evidence. The most cost-effective instruments were QPPS, NORPEQ and I-PAHC. All instruments demonstrated good or excellent acceptability, except QPP. Several instruments (HCAHPS, SIPES, NORPEQ, I-PAHC and PPQ) were rated as good for educational impact.

**Table 6:** Results of Beattie and Murphy instrument utility matrix

Instrument	Primary purpose	Validity			Reliability			Cost efficiency	Acceptability	Educational impact
		Content/theoretical development	Construct (structural, cross-cultural)	Criterion validity	Internal consistency	Other reliability	Was the correct error source investigated?	Rating	Rating	Rating
HCAHPS	National comparisons	*(−)	****(?)		****(+)	***/****(^+^_?_)	Y	*	***	***
QPP	Quality improvement	****(+)	***(^+^_?_)	*(+)	****(+)		Y	**	**	**
QPPS	Quality improvement			****(+)	****(+)		P	***	***	**
PPE-15	National performance indicators	****(+)	****(+)	***(+)	***/****(+)		P	**	***	**
NHSIP	National performance indicators	****(+)			**(?)		N	*	***	**
SIPES	National comparisons	****(+)			*(+)		N	*	****	***
HKIEQ	National comparisons	****(+)	**(^+^_?_)		**(+)	**(+)	Y	*	***	**
PEQ	Quality improvement and national surveillance	***(+)	**/***(^+^_?_)		**(+)	**(+)	Y	**	****	*
NORPEQ	Cross-national comparisons in Nordic countries	***(+)	***(+)		**(+)	***(+)	Y	***	****	***
I-PAHC	Quality improvement in low-income settings	****(+)	***/****(^+^_?_)		****(+)		P	***	***	***
PPQ	Local quality improvement	****(+)	****(+)		****(+)		P	**	****	***

Ratings of study quality: *poor, ** fair, ***good, ****excellent. Ratings of measurement results: (+) positive rating, (−) negative rating, (?) indeterminate rating, (^+^_?_) mixed. Correct source of error: Y yes, N no, P partial

## Discussion

To our knowledge, this is the first systematic review to identify and critique the utility of instruments aiming to measure patient experience of hospital quality. We found 11 international instruments measuring the patient experience of hospital care, while we dismissed numerous measuring patient satisfactions. We critiqued utility from a wide perspective, using international standards where they were available and devising additional criteria where needed.

Reassuringly, all instruments reported some psychometric testing and published information on other aspects of utility. Similar literature reviews have found that studies do not report sufficient psychometric information to enable critique, although this has improved over the last 10 years [7, 21]. We found enough reported psychometric information to critique the retained instruments, although some missing data may have resulted in studies being apportioned lower scores for study quality.

Of course, validity and reliability are not ‘all or nothing’ concepts; rather, they are a matter of degree. Evidence of validity tends to be cumulative, as each new study provides further confirmation of the ability of an instrument to measure patient experience of hospital quality care. As validation develops over time, it is important not to dismiss newer instruments with only some validation. The reliability of an instrument is also strengthened over time as developers refine the tool and identify ways in which to reduce the margin of error, such as the establishment of a training manual and, of course, developments in psychometrics.

While the longevity of instruments is an identified strength, there should also be a note of caution. Well-established instruments may rely on historical data to establish theories and concepts of quality of hospital care. What constitutes Quality from the Patients’ Perspective is likely to shift over time [4]; therefore, we suggest that elements of hospital care which are important to patients are re-explored at least every few years, to re-ensure continued instrument validity. We also found evidence of items being added to instruments to fit the current healthcare policy context [6, 46]. While this seems reasonable, there is a risk that an instrument becomes a measure of healthcare policy implementation as opposed to measuring the patient experience of the quality of hospital care. Conducting interviews or surveys to assess the impact of additional items addressing policy aims should also ensure that such changes do not alter the overall validity of questionnaire content from the patient’s perspective. We found extensive work in terms of theoretical and conceptual development of instruments in this area, which is necessary for an elusive and evolving concept of quality of health care.

We found no studies assessing the ability of an instrument to detect change over time in the construct to be measured, otherwise known as responsiveness [15]. This was surprising given that one of the main uses of patient experience instruments is to measure hospital care quality for evaluation of local improvement work. This review highlights both the need for and the current gap in studies assessing responsiveness of these instruments.

This systematic review highlights that there is no ‘one-size-fits-all’ approach in selecting an instrument to measure the patient experience of hospital quality of care. Rather, there are a range of instruments available with varying strengths and limitations of instrument utility. Instrument choice will, therefore, be dependent upon a number of factors, specifically the purpose for which the data will be used, available resource and local context. For example, where an instrument is to be used for high stakes purposes (perhaps attached to a financial incentive, public league tables or an outcome measure in a research study), an instrument with high reliability should be selected, such as HCAHPS. However, high costs in terms of resource would need to be accepted as HCAHPS requires compliance with standardised sampling, data collection and statistical expertise to analyse the data. Alternatively, if an instrument is to be used to measure the effectiveness of local quality improvement work, then QPPS may be the instrument of choice, as it rated good for user acceptability and cost efficiency. Similarly, but in a low-income setting, I-PAHC could be a useful instrument as it has scored ‘good’ and ‘excellent’ in all dimensions of instrument utility. Also, brief instruments, such as QPPS or PPE-15, may be used as screening instruments to determine a sample for more detailed exploration.

Context is also important, particularly in relation to theoretical development and content validity. For example, if work has been carried out to determine what quality of hospital care means to a local population, as with SIPES in Scotland, then this would be the instrument of choice in Scotland in terms of its content validity. Where instruments are utilised in other countries, studies of cross-cultural validity should be conducted before instrument use.

As with all literature reviews, our findings are dependent upon the quality of detail available in the published literature. There are risks that unpublished instruments have been missed. While our literature search did not include the EMBASE database for pragmatic reasons, we did conduct a thorough search of MEDLINE, CINHAL and PsychINFO, as well as specialist databases in the field of patient experience. We also acknowledge that only 10 % of the inclusion criteria was independently checked by two reviewers. Despite checking secondary references, we found no other instruments meeting our inclusion criteria.

Also, there is a possibility that included instruments have been harshly critiqued. We used the COSMIN criteria which reduces scores for methodological quality when insufficient information is available and applies the ‘lowest score counts’ for an overall score [3]. Some psychometric studies may have only been rated as poor or fair on one item response, subsequently giving a low overall rating. However, a design strength of the COSMIN four-point rating scale was to ensure that only fatal flaws are categorised as poor. Therefore, some item responses cannot be categorised as poor. For example, some checklists determine whether or not the percentage of missing items was reported. Responses are either ‘yes’ or ‘no’. A response of ‘no’ could still achieve a ‘good’ quality rating as this question did not offer a ‘poor’ response option. While having missing items is not regarded as good practice, COSMIN developers determine that the overall quality of the study could still be good or excellent [51]. We limited bias by making reasonable attempts to contact instrument developers for further information and complete scoring independently before arriving at definitive results.

Using the criteria from Terwee et al [50] for results of measurement properties offered a rigorous, equitable and transparent critique of study results. Some instruments may have just fallen below the criteria set and therefore been rated as a negative. That is not to say the instrument cannot be used; rather, some caution should be applied when considering instrument selection. Depending on the purpose of the instrument, lower levels of reliability may have been acceptable; however, the cut-off point needed to be set somewhere.

There were also some psychometric results which did not fit the Quality Criteria for Measurement Properties’ results [50], such as studies which used structural equation modelling, which were subsequently categorised as indeterminate. Applying the quality criteria was extremely time-consuming; for example, some studies took several hours. Some criteria required to be more explicit; for example, the criteria for structural validity required factors to explain more than 50 % of variance. It was unclear whether 50 % was required for each factor or total factors. We used total factors and reached decisions on anomalies through consensus discussion.

We do not suggest that the additional dimensions of utility are definitive; rather, this paper offers a starting point of a method to critique these additional, but fundamental, aspects of instrument use. Although offering a degree of face validity, further work is required to determine application to instruments measuring other constructs. A working manual would also provide explanatory guidance for other users. As well as instrument selection, the matrix can also be used to identify research gaps for existing instruments, for example, further validity testing for the SIPES instrument or reliability studies for NHSIP. Instrument development should start with a sound theoretical development of what constitutes Quality from the Patients’ Perspective. New instruments may be necessary if there are revised theoretical and conceptual developments of what constitutes quality of hospital care. Advances in how to quantify patient experience may also necessitate the development of new instruments.

## Conclusions

Patient experience data could be used to drive improvements in hospital care at national, local and healthcare team levels. To date, there are a range of instruments available to measure the patient experience of hospital quality care. Clinicians, managers, policy makers and researchers need to select patient experience instruments which are fit for purpose. This study aims to aid this choice by providing a framework to allow consideration of a wide perspective of the utility of instruments. Users can weigh the importance of each dimension, depending on the purpose of data collection, thus aiding instrument choice. Selecting the right patient experience instrument for the right purpose can aid improvements in hospital quality of care.

## Additional files

Additional file 1:
**PRISMA (2009) Checklist.** This file contains a checklist of the criteria necessary for reporting a systematic review, detailing where specific criteria are covered in the paper. Additional file 2:
**Search results.** This file contains the search strategy conducted in MEDLINE and results of all database and grey literature searching.

## Abbreviations

CINAHL: Cumulative Index to Nursing and Allied Health Literature
COSMIN: Consensus-based Standards for the Selection of Health Measurement Instruments
HCAHPS: Hospital Consumer Assessment of Healthcare Providers and Systems
HKIEQ: Hong Kong Inpatient Experience Questionnaire
I-PAHC: Patient Experiences with Inpatient Care
MEDLINE: Medical Literature Analysis and Retrieval System Online
NHSIP: National Health Service Inpatient Survey
NORPEQ: Norwegian Patient Experience Questionnaire
PEQ: Patient Experience Questionnaire
PPE-15: Picker Patient Experience Questionnaire
PPQ: Patient Perceptions of Quality
PsychINFO: Psychological Information
QPP: Quality from the Patients’ Perspective
QPPS: Quality from the Patients’ Perspective Shortened
SIPES: Scottish Inpatient Patient Experience Survey
