# Supplementary material for: Instruments to measure patient experience of healthcare quality in hospitals: a systematic review
Source: Syst Rev. 2015 Jul 23;4:97. doi: 10.1186/s13643-015-0089-0 (PMC4511995; doi:10.1186/s13643-015-0089-0)
Supplement: Additional file 2: — Search results. This file contains the search strategy conducted in MEDLINE and results of all database and grey literature searching. [file 13643_2015_89_MOESM2_ESM.docx]

**Ovid MEDLINE(R)** **Search Strategy**

| **#** | **Advanced Search** |
| --- | --- |
| 1 | Patient-Centred Care/ |
| 2 | exp *Quality Indicators, Health care/ |
| 3 | is.fs. |
| 4 | *”Process Assessment (Health Care)”/ |
| 5 | *”Health Care Surveys”/is (Instrumentation) |
| 6 | patient-reported.mp. |
| 7 | *”Questionnaires”/st (Standards) |
| 8 | quality of care.mp. |
| 9 | health care surveys/ or questionnaires/ |
| 10 | patient experience.mp. |
| 11 | *”Outcome Assessment (Health Care)” / |
| 12 | *”inpatients”/ |
| 13 | is.fs. or measure*.mp. or validation.mp. |
| 14 | inpatients/ |
| 15 | Secondary Care/ |
| 16 | hospital*.mp. |
| 17 | (acute adj (service* or care or setting*)).mp. |
| 18 | (patient* adj3 experience*).mp. |
| 19 | (quality* adj3 (care or healthcare)).mp. |
| 20 | 1 or 10 or 18 |
| 21 | 14 or 15 or 16 or 17 |
| 22 | 5 or 13 |
| 23 | 20 and 21 and 22 |
| 24 | 2 or 8 or 19 |
| 25 | 23 and 24 |
| 26 | (patient* adj2 (perspective* or opinion* or experience*)).mp. |
| 27 | 25 and 26 |

**Search Results 30^th^ November 2013**

| **Database** | **Results** |
| --- | --- |
| MEDLINE (R) | 275 |
| CINHAL | 719 |
| Psych Info | 163 |
| **Total Database results** | **1157** |
|  |  |
| Health Foundation Website <http://www.health.org.uk/> | 10 |
| CAHPS website <https://cahps.ahrq.gov/> | 3 |
| PROQOLID The Patient-Reported Outcome and Quality of Life Instruments Database. All generic instruments searched <http://www.proqolid.org/> | 0 |
| MCJ & Supplement | 10 |
| Scottish Government website/emails | 3 |
| Picker website/emails | 5 |
| Colleague | 1 |
| Health Service Journal | 5 |
| Secondary references | 21 |
| **Total Identified Through Other Sources** | **56** |
|  |  |
| Minus duplicates | 215 |
|  | **1000** |
